# 10 MHz Thin-Film PZT-Based Flexible PMUT Array: Finite Element Design and Characterization

**DOI:** 10.3390/s20154335

**Published:** 2020-08-04

**Authors:** Jeong Nyeon Kim, Tianning Liu, Thomas N. Jackson, Kyusun Choi, Susan Trolier-McKinstry, Richard L. Tutwiler, Judith A. Todd

**Affiliations:** 1 Department of Engineering Science and Mechanics, The Pennsylvania State University, University Park, PA 16802, USA; juk252@psu.edu; 2 School of Electrical Engineering and Computer Science, The Pennsylvania State University, University Park, PA 16802, USA; twl5075@psu.edu (T.L.); tnj1@psu.edu (T.N.J.); kxc104@psu.edu (K.C.); 3 Department of Materials Science and Engineering, The Pennsylvania State University, University Park, PA 16802, USA; stmckinstry@psu.edu; 4 Applied Research Laboratory, The Pennsylvania State University, University Park, PA 16802, USA; rlt1@psu.edu

**Keywords:** flexible 2D PMUT arrays, finite element analysis, PZFlex, virtual prototyping, pulse-echo/spectral response, surface displacement, bandwidth, crosstalk, beam profiles

## Abstract

Piezoelectric micromachined ultrasound transducers (PMUT) incorporating lead zirconate titanate PbZr_0.52_Ti_0.48_O_3_ (PZT) thin films were investigated for miniaturized high-frequency ultrasound systems. A recently developed process to remove a PMUT from an underlying silicon (Si) substrate has enabled curved arrays to be readily formed. This research aimed to improve the design of flexible PMUT arrays using PZFlex, a finite element method software package. A 10 MHz PMUT 2D array working in 3-1 mode was designed. A circular unit-cell was structured from the top, with concentric layers of platinum (Pt)/PZT/Pt/titanium (Ti) on a polyimide (PI) substrate. Pulse-echo and spectral response analyses predicted a center frequency of 10 MHz and bandwidth of 87% under water load and air backing. A 2D array, consisting of the 256 (16 × 16) unit-cells, was created and characterized in terms of pulse-echo and spectral responses, surface displacement profiles, crosstalk, and beam profiles. The 2D array showed: decreased bandwidth due to protracted oscillation decay and guided wave effects; mechanical focal length at 2.9 mm; 3.7 mm depth of field for -6 dB; and -55.6 dB crosstalk. Finite element-based virtual prototyping identified figures of merit—center frequency, bandwidth, depth of field, and crosstalk—that could be optimized to design robust, flexible PMUT arrays.

## 1. Introduction

The development of a miniature, ultrasound, finger vein imaging sensor for personal authentication in mobile phones, tablet and laptop computers, and personal electronic devices is of interest. Currently, fingerprint-based biometrics are used for this purpose, however, this method is fallible as fingerprints can be stolen or unclear, due to injury or chemical exposure. Finger vein structures are as unique to individuals, as fingerprint patterns are buried and protected under the skin. Consequently, they can provide a more secure, but still accessible, means of personal authentication.

Such devices would be enabled by thin-film, PbZr_0.52_Ti_0.48_O_3_ (PZT)-based piezoelectric micromachined ultrasound transducers (PMUTs). Mina et al. [1] designed and fabricated 1D and 2D PZT microelectromechanical system (MEMS) ultrasonic transducers for operating frequencies above 30 MHz. Griggio et al. [2] successfully demonstrated a 35 MHz miniaturized 15 × 32 ultrasound array consisting of 130-µm diameter micromachined diaphragms, providing the design, fabrication and experimental measurements of the array transducers. Zhu et al. [3] demonstrated a 100 MHz, kerfless, 32-element, 1D ultrasound array with a 15-µm thick, 1 × 1 cm area, PZT film. Qiu et al. [4] discussed fabrication, electrical integration, and electrical and acoustic measurements of PMUT arrays for sensing, actuation and imaging. Nakazawa et al. [5] fabricated eight-element, 200 µm pitch and 32-element, 20-µm pitch, 1D arrays based on a polyurea film, and experimentally measured the resonance frequencies at 30 MHz, 65 MHz, and 100 MHz. Cheng et al. [6] reported a 2D PMUT array consisting of 20 diaphragms, with each diaphragm having 60 µm diameter, and showed its resonance frequency at 8 MHz with 62.5% bandwidth. 9.5 kPa of acoustic pressure was measured by a hydrophone (HGL-0085, Onda, Inc,.) at 7.5 mm distance from the PMUT array surface. The PMUT array demonstrated particle manipulation of 4-µm silica beads at driving frequencies up to 60 MHz. Unique bead patterns were observed at different driving frequencies and levitation planes were found above the 30 MHz driving frequency. Sadeghpour et al. [7] presented a silicon-on-insulator (SOI)-based bendable PMUT array. Five 3 × 3 PMUT arrays with 426 kHz resonance frequency, with each PMUT having 410-µm diameter and 6-µm thickness with a 1-µm PZT layer, were fabricated on five Si islands. Six Si islands, including the five with PMUT arrays, were connected by silicon springs to wrap a 5 × 5 × 5 mm cube. Most of the studies on PZT thin/thick film-based, high-frequency PMUTs have the following commonalities: (a) a focus on fabrication methodologies; (b) less weight on beam propagation and pressure field modeling; (c) mostly limited to 1D linear arrays; and (d) only a few 2D array studies demonstrated successful transmission of the ultrasound field. A review of prior research reveals the need for comprehensive modeling of PMUT devices to ensure appropriate acoustic and mechanical characteristics, not only of a desired center frequency and wide bandwidth, but also of targeted focal distance, depth of focus, beam directionality, suppressed modal distortion, and minimized crosstalk.

Modeling approaches include analytical methods based on classical boundary condition theories that include: simply supported circular or rectangular membranes or plates; edge-clamped circular or rectangular membranes or plates; equivalent circuit model analyses. Analytical modeling was usually applied to predict resonance frequencies and deflections of simple transducer structures. As transducer structures evolved in complexity, with multiple layers and then arrays, numerical modeling approaches, mostly finite element methods, were applied to analyze and predict overall system functionality. In 1973, Denkmann et al. [8] analyzed metal–ceramic transducers coupled to resonator systems incorporated in an acoustic chamber, a port, and a porous plug, which are analogous to stiffness, mass, and damping in a mechanical system. In their modeling work, they applied classical boundary value techniques, simple direct variation procedures, and finite element methods. Voltage outputs from different models, unimorph and bimorph, with and without acoustic chambers and ports under different clamping diameters, were calculated and compared. Even though this work is not for micromachined transducers, it is worth mentioning since the coupling of metal–ceramic structures is a base architecture for PMUTs. In 1997, Bernstein et al. [9,10] presented a unimorph, thin-film, PZT-based PMUT and 8×8 arrays of the PMUT for acoustic imaging in the frequency range 0.5–4 MHz. To predict the resonance frequency, they applied ideal circular bimorph analysis to a simply supported and clamped edge circular plate, with one-side water loading and with no water loading. Finite element analyses were used to compute resonance frequencies, stress distributions, and static sensitivities for square- and rectangular-shaped diaphragms in the complex multiple layered structures. Measured sensitivities and finite element calculations were found to agree within 10 dB. In the same year, Percin and Khuri-Yakub [11] demonstrated a thin film, ZnO-based, ring-shaped, PMUT 2D array and a fully supported circular membrane PMUT 2D array. Finite element modeling was applied to optimize individual PMUT element resonance frequencies, input impedances, and normal surface displacements. Several iterations of ANSYS calculations found that the maximum displacement of a ring-shaped ZnO PMUT on a silicon nitride (Si_3_N_4_) membrane occurred when the ZnO ring had an inner diameter of 30 µm. ANSYS calculation of the resonance frequency, 3.46 MHz, of the structure agreed well with that measured experimentally in vacuum, 3.07 MHz. In 1999, Bernstein et al. [12] expanded their PMUT array research to a 3 MHz, 16 × 16 2D array, with sol-gel PZT on zirconia (ZrO_2_) and silicon dioxide (SiO_2_). They stated that sensitivity could be improved by 30 dB using in-plane polarized PZT compared to through-thickness polarized PZT, though at the expense of higher drive voltages. Lumped element equivalent circuit model analyses predicted the optimum thickness of the wafer to be 295 µm for 1 and 3 MHz resonance frequencies. Finite element analyses for different designs—four circular diaphragms in 0.4 × 0.4 mm unit-cells—were conducted using ANSYS software. Pressures at distances of 0, 50, and 100 µm from the devices were calculated as a function of frequency. Transmit response and receiving sensitivities were predicted. 

In 2004, Akasheh et al. [13] demonstrated PMUTs in the frequency range 2–10 Mz in water. The PMUTs were structured on a Si membrane with thicknesses of 1–5 μm, and widths of 30–150 μm. A SiO_2_ layer, 100–600 nm thick, was deposited on the Si membrane. A 200-nm thick titanium (Ti)/Pt bottom electrode, PZT active layer, and 200-nm thick gold (Au) top electrode were deposited on the SiO_2_ layer, in that order. They configured a three top electrode structure—one center electrode to excite the PZT and two side electrodes symmetrically aligned about the center electrode. The effect of the membrane width on resonance frequency was investigated using PZFlex, a finite element method software. The coupling coefficient and acoustic impedance was examined. In 2008, Choi et al. [14] published a modeling study of a PMUT with a high length to width aspect ratio. They designed and inspected 20 different dimensions of single-element PMUTs having aspect ratios ranging from 5:1 to 23:1. A 1D composite beam model was applied with an equivalent circuit model to correlate the equivalent circuit components with the structural parameters and predict the characteristics of the PMUTs. They discovered that the model became more realistic when the effect of residual stress was included and concluded that the effective coupling coefficient was not just a function of the membrane width but also depended on the relative ratio of the electrode and membrane widths. In 2012, Sammoura and Kim [15] presented an electric circuit model development for a circular bimorph PMUT. The bimorph PMUT was comprised of two layers of the same thickness and same radius piezoelectric material sandwiched between three thin electrodes. The two piezoelectric layers were biased with the same magnitude of electric potential but opposite polarities. The equivalent circuit model consisted of an elastic part and a mechanical part which were coupled through a transformer element. Electric input impedance and the average displacement of a simply supported bimorph PMUT model were predicted using the equivalent circuit network. They found that the derived electric circuit model provided better PMUT design, characterization and performance predictions. In the same year, Sammoura et al. [16] reported analytical modeling of a unimorph PMUT with multiple layers and a circular top electrode surrounded by an arbitrary number of outer rings. Classic clamped plate theory was applied to derive equations of motion for axisymmetric bending incorporating residual stress, external pressure, and voltage input. The equation of motion was solved using Green’s function. 

In 2013, Yang et al. [17] introduced PMUT arrays using the reverse bonding of SOI and release of the backside bulk Si technique, and showed high element density and low crosstalk. For the device performance prediction, finite element simulation was conducted using ANSYS software. The finite element model from the bottom to top consisted of a Si substrate, 0.3-μm thick passive SiO_2_ layer, 0.2-μm thick Pt bottom electrode, 2-μm thick PZT layer, and 0.2-μm thick Pt top electrode. The planar dimensions of the modeled element were 100 μm in both length and width. The impedance spectrum and vibration amplitude were then analyzed. In 2016, Lu and Horsley [18] reported high fill-factor PMUT array development using cavity SOI wafers. PMUTs with PZT and aluminum nitride (AlN) piezoelectric layers, of diameters from 25 to 50 μm, were fabricated showing corresponding center frequencies from 13 to 55 MHz. Finite element analyses were conducted to find the optimal receiving performance in terms of piezoelectric active layer thickness. Directivity patterns for PMUTs with diameters of 30, 100, and 200 μm were theoretically calculated and plotted, showing that smaller diameters gave more spherical spreading patterns. Finite element calculations were carried out to find the optimal radius of the top electrode. The radius where the sum of radial and tangential stresses was zero on the PMUT surface, which is the optimal electrode radius in receiving mode, was found to be about 0.707 times the PMUT surface radius. Resonance frequencies in air were calculated using COMSOL Multiphysics for PMUT cell radii from 12.5 to 25.0 μm, then compared with experimental measurements. Static displacement sensitivities in air were also calculated using the finite element method and measured experimentally for different PMUT cell radii. 

More recently, in 2017, Dangi and Pratap [19] reported a system level modeling study to develop parametric design maps of PMUTs. The behaviors of PMUTs, plate, plate and membrane mixed, and membrane were discussed in terms of the non-dimensional ratio between residual tension to flexural rigidity. PMUT was investigated as a transmitter, a receiver, and a transceiver applying a lumped model approach, and expressions for performance parameters for each case, such as deflection at the center, and the charge generated across a PMUT, were found. A plate type PMUT having a 10-µm thick Si passive layer, and 650-nm thick PZT layer with 500-µm plate radius, and membrane type PMUTs having a 300-nm thick SiO_2_ passive layer and 1-µm thick PZT layer with various membrane radii, 100, 150, 200, and 500 µm, were fabricated. Both types had a Ti (20-nm thick)/Pt (150-nm thick) bottom electrode, and a chromium (Cr) (10-nm thick)/Au (150-nm thick) top electrode. Resonance frequencies for the membrane type PMUTs were analytically calculated and compared with experimental results for the first three modes with errors from 0.4% to 13.4%. Acoustic pressure of the plate type PMUT in air was theoretically estimated at distances from 50 to 500 mm and showed good agreement with those experimentally measured. In 2020, Massimino et al. [20] presented a finite element modeling study of PMUTs in which fabrication induced residual stresses and packaging influences were considered. A 4 × 4 PMUT array with each cell consisting of a 308-µm radius, 1.06-µm thick PZT layer on top of a 440-µm radius, 4.25-µm thick Si layer was modeled using ANSYS. A 400-µm deep, 440-µm radius air-filled cavity was placed beneath the diaphragm. The numerically computed static deformation due to the residual stresses agreed well with the white-light interferometry measurement. The model analyses with and without a protective package, and with various package designs, including a center hole with different diameters, holes at the center of each transducer, and randomly distributed holes, suggested that sound pressure level (SPL) and acoustic propagation were governed by the package vibration. Additional detailed reviews of previous PMUT studies and their modeling approaches have been discussed by Kim [21].

In 2017, Liu et al. [22,23] developed a process to remove the PMUT from an underlying rigid Si substrate and enabled the formation of micromachined curved arrays. The research described in this paper aims to improve the design of flexible PMUT arrays using an explicit, time-domain, finite element method software, PZFlex [24,25]. A 10 MHz, 2D array, PMUT device, consisting of 256 (16×16) circular unit-cells working in 3-1 mode, was designed and characterized in terms of electrical impedance, pulse-echo and spectral responses, mode shapes, surface displacement profiles, beam profiles and crosstalk. Details of the design, characterization procedures, and results will be discussed.

## 2. Unit-Cell Design

**Figure 1:**
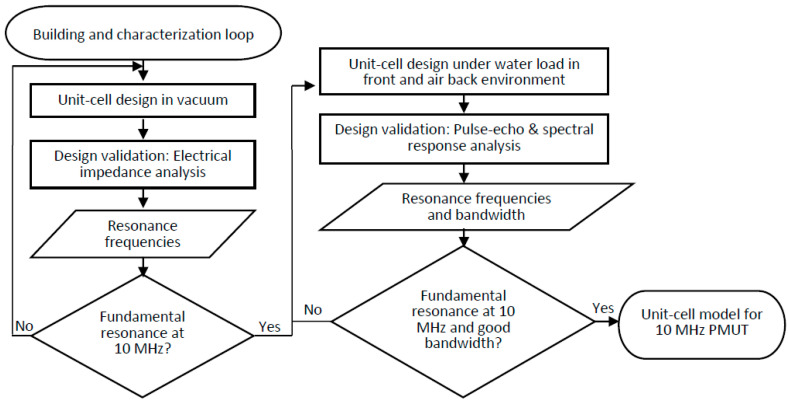
Building, characterization and validation loops for the piezoelectric micromachined ultrasound transducers (PMUT) unit-cell design.

Virtual prototyping of the unit-cell design using PZFlex began with building, characterization and validation loops shown in Figure 1. This procedure began by searching for a unit-cell geometry for a targeted fundamental resonance frequency of 10 MHz in vacuum. Finite element analysis is more efficient in terms of time in vacuum than in elastic media, especially when multiple iterations and parametric studies are implemented. Plotting electrical input impedance revealed the resonance frequencies of the structure. Once a unit-cell geometry with a desired fundamental resonance was discovered, a more realistic environment could be applied. In this study, a water load in front of the unit-cell was applied as a wave propagation medium and air-backing was added to reproduce the experimental measurement environment. Pulse-echo and spectral response analyses were conducted to determine the ring-down characteristics, center frequencies, and bandwidths of the unit-cell models. Finally, two circular unit-cell prototypes were investigated.

### 2.1. Unit-Cell Prototype 1

#### 2.1.1. Dimensions and Material Properties

**Figure 2:**
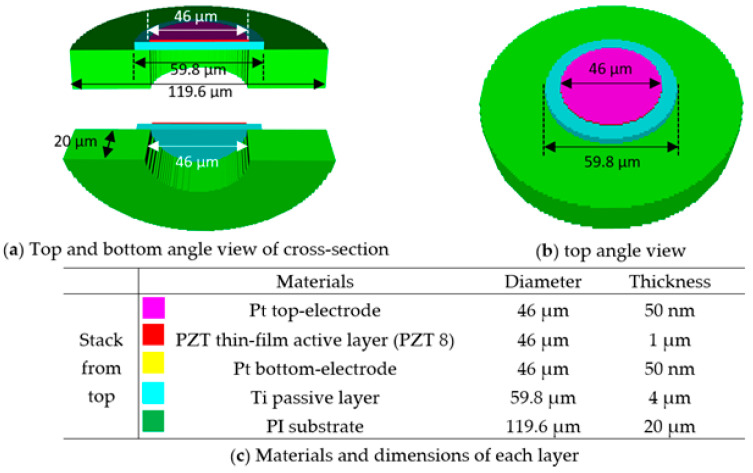
10 MHz circular unit-cell prototype in (**a**) top and bottom cross-section, (**b**) top angle view and (**c**) materials and dimensions of each layer.

A circular unit-cell consisting of a 50-nm thick Pt top electrode, 1-µm thick active PZT film, 50-nm thick Pt bottom electrode, and 4-µm thick Ti passive layer, was designed. All the layers were deposited on a 20-µm thick PI substrate in which a vacuum cavity, for computational efficiency, was concentrically placed, as shown in Figure 2. The diameters of the active PZT layer, the passive Ti layer, and the cavities were 46, 59.8, and 46 µm, respectively. The diameter and thickness of the Ti layer was initially predicted from classical plate theory, which will be discussed in Section 2.1.3. Multiple simulation iterations were made to optimize the diameters of the Ti layer, the PZT layer, Pt electrodes, and the cavity to achieve the desired resonance frequency. The PI substrate was 119.6 µm in diameter and both Pt electrodes had the same diameter as the PZT active layer. Figure 2 shows the unit-cell with its dimensions. 

**Figure 3:**
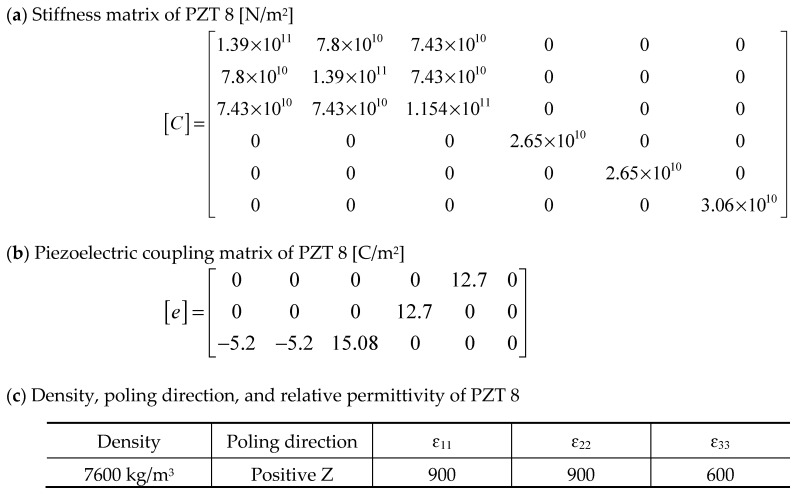
(**a**) Stiffness, (**b**) piezoelectric coupling matrices, and (**c**) density, poling, and relative permittivity of PbZr_0.52_Ti_0.48_O_3_ (PZT) 8 used for the modeling.

PZT 8, a high-power, low dielectric loss, high mechanical strength, and good stability piezoelectric ceramic (Piezo Hannas, Wuhan, China) was selected as the piezoelectric material to model the PZT thin-film active layer. It is important to note that there has not yet been complete characterization of the elastic and piezoelectric properties of PZT thin films. Moreover, the complexities of the microfabrication process may alter the original properties of PZT thin films, and thus the properties may not be optimal for predicting the performance of fabricated thin-film PZT-based PMUTs [26]. Figure 3 displays the stiffness (*C*), piezoelectric coupling (*e*) matrices, density, poling direction, and relative permittivity (ɛ_11,_ ɛ_22,_ and ɛ_33_) of PZT 8 used in the model.

The properties of Pt, Ti, and PI [27,28] applied in the finite element analysis are listed in Table 1.

The attenuation coefficients of PI were calculated using the Damping Tool [29] (p.89) in PZFlex 2018 based on its mechanical properties displayed in Table 1.

#### 2.1.2. Validation with Electrical Input Impedance in Vacuum

**Figure 4:**
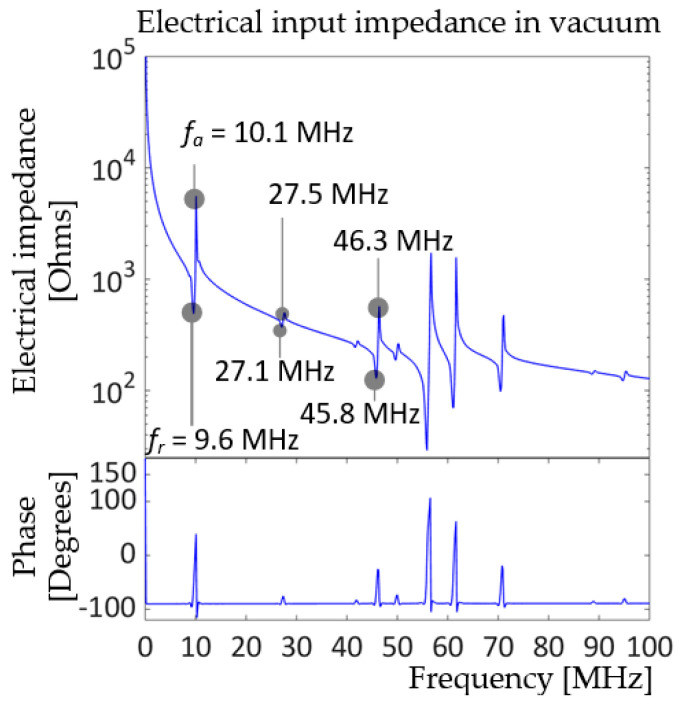
Electrical input impedance in vacuum of unit-cell prototype driven by 2 V_pp_, 100 kHz–500 MHz sweep signal with 50 Ω resistor in the circuit.

The model was validated for its resonance and anti-resonance frequencies by plotting the electrical input impedance in vacuum. The model was driven by a peak-to-peak bipolar voltage of 2V_pp_, 100 to 500 MHz sinusoidal, not enveloped, sweep signal, to make sure that there were no resonances below the targeted fundamental resonance frequency, 10 MHz. Figure 4 shows the electrical input impedance plot of the model with its fundamental resonance (*f_r_*) and anti-resonance (*f_a_*) frequencies at 9.6 and 10.1 MHz, respectively, very close to the targeted resonance. Higher resonances were found near 27 and 46 MHz. Due to the complexity of the structure, double-layered diaphragm and compliant substrate, higher resonances were not exact harmonics of the fundamental.

#### 2.1.3. Validation with Classic Plate Theory

An analytical approach was taken to validate the fundamental resonance frequency. Equation (1) presents the fundamental resonance frequency of a clamped circular plate in terms of its dimensions and mechanical properties [30] (pp. 107–109)

(1) $$f_{1}=\frac{g_{1}^{2}}{2\pi }\frac{d}{\sqrt{12}}{\left(\frac{Y}{\rho (1-\sigma^{2})}\right)}^{1/2}=0.47\frac{d}{a^{2}}{\left(\frac{Y}{\rho (1-\sigma^{2})}\right)}^{1/2},$$

where *d* and *a* are the thickness and radius of the plate, respectively. *Y*, *ρ*, and *σ* are mechanical properties, the elastic modulus, density, and Poisson’s ratio, respectively. In Equation (1), the product of *g_1_* and *a* is the first argument of the Bessel function (*J_m_*) and the modified Bessel functions (*I_m_*) of the first kind of order m, which satisfies Equation (2).

(2) $$J_{0}(g_{n}a)/J_{1}(g_{n}a)=-I_{0}(g_{n}a)/I_{1}(g_{n}a),$$

The first four values of *g_n_a* are 3.20, 6.30, 9.44, and 12.57, with *n* = 1, 2, 3, and 4, respectively. With increasing *n* values, *g_n_a* becomes *nπ*. The detailed derivation can be found in Kinsler et al. [30]. (pp. 107–109). Applying a 4-µm thick, 29.9-µm radius Ti plate with *Y, ρ,* and *σ* equal to 113.8 GPa, 4510 kg/m^3^, and 0.33 [31], respectively, to Equation (1) gives the lowest natural frequency, *f_1_* = 11.2 MHz. This value differs from that of the finite element model, where *f_r_* = 9.6 MHz and *f_a_* = 10.3 MHz, since the analytical approach considered only a single, perfectly clamped Ti plate, while the finite element model consisted of two layers on a flexible substrate. Furthermore, the flexible PI substrate caused the effective area of the vibrating plate to become larger than that of the given plate theory. In addition to plate theory, clamped membrane theory is often used to predict the resonance frequency of thin-film PZT devices. The major difference between the two is that the restoring force of a membrane arises entirely from the applied tension, while that of a thin plate results from the stiffness of the diaphragm [30] (p. 107). Hong et al. [32] reported an in-depth study which applied membrane theory to the analysis of circular piezoelectric diaphragm vibration. Please note that this study treated the thin-film PZT and elastic passive layers as thin plates rather than membranes because in finite element studies on membranes, the thickness of membranes tends to be either not considered in meshing or meshed with only a single layer [33,34,35,36,37,38]. Kim [21] conducted model reliability and mesh sensitivity studies of a thin-film, PZT-based structure with different mesh conditions and simulation durations, and concluded that at least three mesh layers in the thin film PZT layer thickness were required to recognize not only motions in the radial or width directions but also those in the thickness direction. This modeling study implemented at least three mesh layers in thickness directions of the elastic active and passive layers to ensure reliability of the models.

### 2.2. Unit-Cell Prototype 2

**Figure 5:**
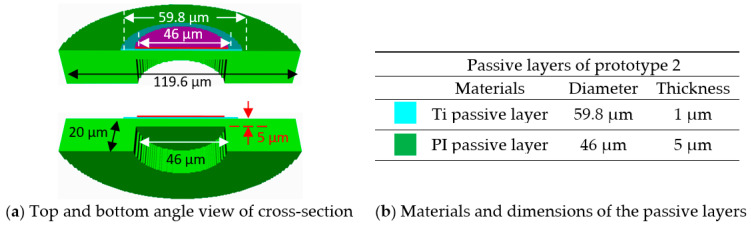
(**a**) Cross-section views and (**b**) passive layers of the unit-cell prototype 2.

The unit-cell prototype 2 was devised to have the same resonance frequency and reduce the complexities of the fabrication process, particularly the long deposition time of a 4-µm thick Ti layer [39]. Figure 5 shows the geometry of the unit-cell prototype 2. 

In Figure 5, the unit-cell prototype 2 has two passive layers—a 1-µm thick Ti layer beneath the PZT active layer, and a 5-µm thick PI layer underneath the Ti layer. The concentric cavity below the diaphragm had 15 µm depth. All other design parameters were the same as those of unit-cell prototype 1.

#### Validation with Electrical Input Impedance in Vacuum

**Figure 6:**
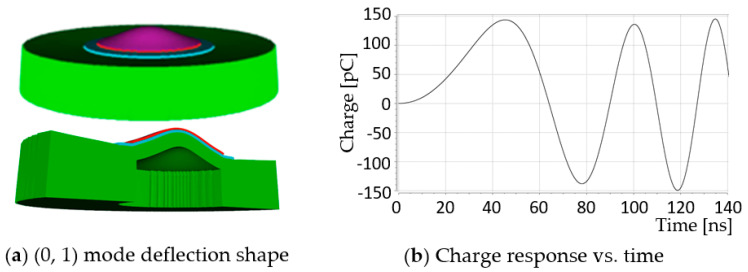
(**a**) (0, 1) mode deflection shape at t = 140 ns., and (**b**) charge response vs. time of the unit-cell prototype 2.

The prototype 2 model was driven by a peak-to-peak bipolar 2 V_pp_ for the frequency range 100 kHz to 500 MHz sinusoidal, not enveloped, and the electrical impedance in vacuum was plotted. Figure 6 displays a snapshot of the PZFlex simulation running in PZFlex.

**Figure 7:**
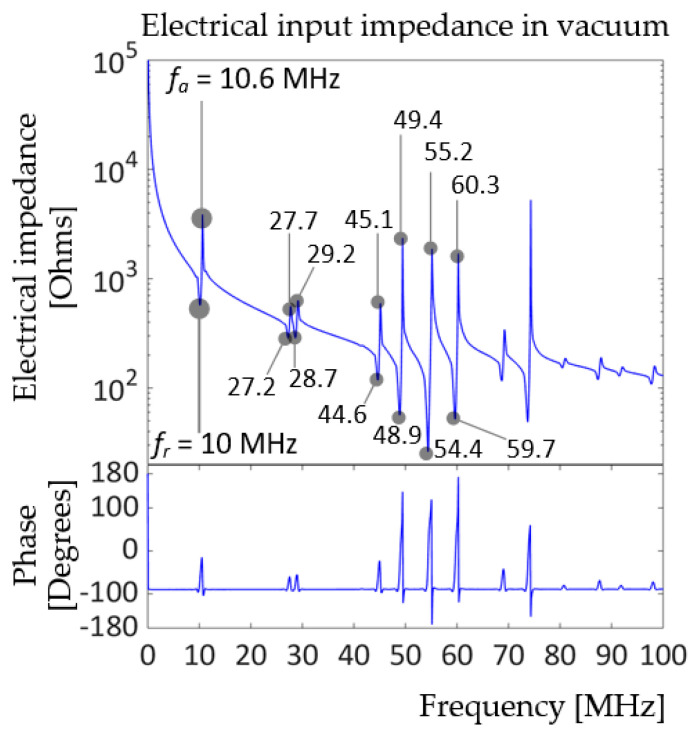
Electrical input impedance of the unit-cell prototype 2 driven by 2 V_pp_, 100 kHz to 500 MHz sweep signal with 50 Ω resistor in the circuit.

Figure 6a shows the (0, 1) mode deflection shape of the prototype 2 at t = 140 ns, and Figure 6b displays the time history of the corresponding charge response. Figure 7 presents the resulting electrical impedance.

The electrical input impedance of the model in Figure 7 is more complicated than that of prototype 1 in Figure 4 due to the addition of a PI passive layer, which is less stiff than the PZT and Ti layers. The elastic modulus of PI is 8.5 GPa [28], while Ti and PZT8 have elastic moduli of 113.8 GPa and at least 74 GPa, respectively, as shown in Figure 3. According to Figure 7, the unit-cell prototype 2 model has its fundamental resonance frequency at 10 MHz and anti-resonance frequency at 10.6 MHz, which satisfies the design goal. Several higher resonances were observed: two small peaks near 28 and 29 MHz, and four consecutive peaks near 45, 49, 55, and 60 MHz. Classic plate theory validation was not carried out for prototype 2, since analytical predictions for complex geometry, i.e., a multilayer plate, particularly one with a compliant layer, tend to be less accurate.

### 2.3. Unit-Cell with Water Load and Air-Backing

**Figure 8:**
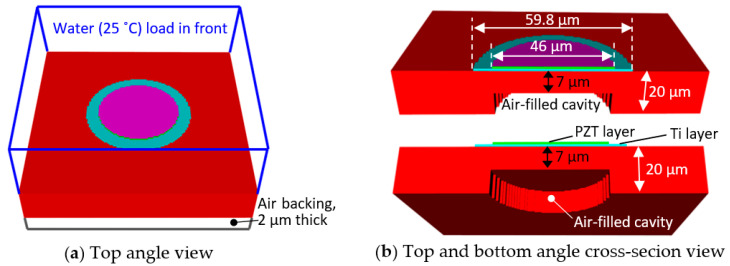
Views of the modified unit-cell model under water load and air-backing.

The unit-cell prototype 2 was selected for the further development as a 2D array due to its advantage in microfabrication. Figure 8 depicts the modified unit-cell model. Please note that in Figure 8, the colors for the layers are: from the bottom, red—PI substrate and passive layer, light blue—Ti passive layer, light green—PZT thin film, and purple—Pt top electrode.

In Figure 8, water at 25 °C was loaded in front of the unit-cell prototype 2 to create the same environment as the experimental measurements. A 2 µm air layer was added in the bottom of the model and the cavity under the diaphragm was filled with air. The PI passive layer thickness was adjusted to 7 µm from 5 µm to compensate for the decrease in the resonance frequency due to the water load acting as mass-loading in the system. The mesh constructions and boundary conditions of the propagation medium and air-backed model are described in Section 2.3.1.

#### 2.3.1. Boundary Conditions and Mesh for Unit-Cell Model

**Figure 9:**
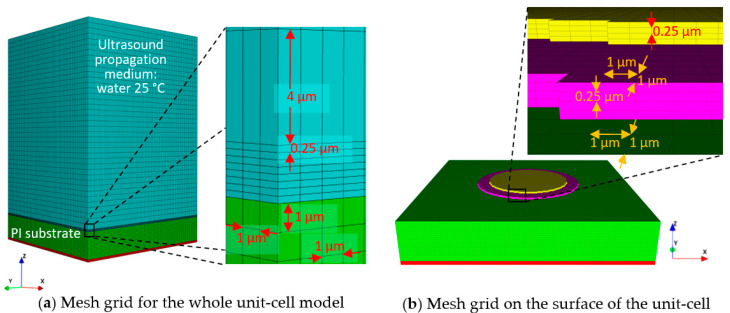
Mesh grid for (**a**) the whole unit-cell model with water load and air-backing, and (**b**) the top surface of the unit-cell.

PZFlex offers conservative rules in meshing and recommends that the aspect ratio of a mesh element should not exceed 1:4 [40]. Figure 9 portrays the model with its mesh grid and dimensions of mesh elements for different sections of the model.

In Figure 9a, the red and green blocks, which are the air-backing and PI substrate, have the same mesh element, 1 × 1 × 1 µm in X × Y × Z coordinates. Above the PI substrate, there are 1-µm thick Ti and 1-µm thick PZT layers, which were meshed with elements having dimensions of 1 × 1 × 0.25 µm in X × Y × Z coordinates. The portion of the water load, which has the same Z coordinates as the Ti and PZT stack, also has the same mesh elements. The remaining water load, which fills the space between the front surface of the unit-cell and the end of the model in the +Z direction, was meshed with elements of 1 × 1 × 4 µm. Figure 9b shows the model without the water load to display the mesh grid at the unit-cell surface. In Figure 9b, the magenta and the yellow blocks are the PZT active layer and Pt top electrode, respectively.

**Figure 10:**
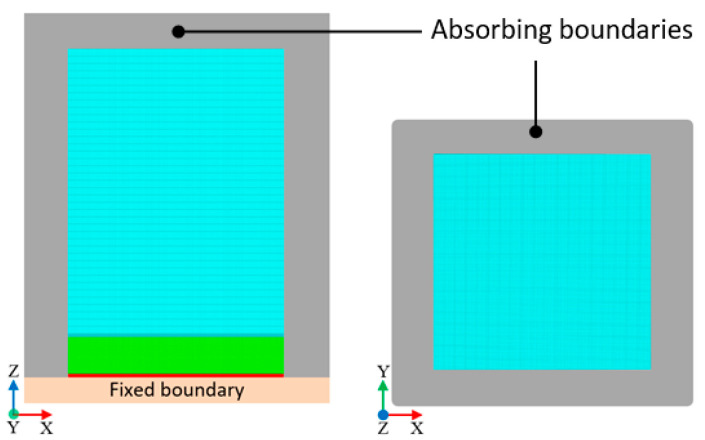
Boundary conditions of the model for three (z-x = z-y, and x-y) orthogonal planes.

Absorbing boundaries were applied to the sides but not the bottom, where an air-backing was applied to prevent reflections. The bottom was treated as a fixed boundary to simulate a stiff bottom cover. Figure 10 illustrates the boundary conditions applied to the model on different planes.

#### 2.3.2. Pulse-Echo and Spectral Responses

**Figure 11:**
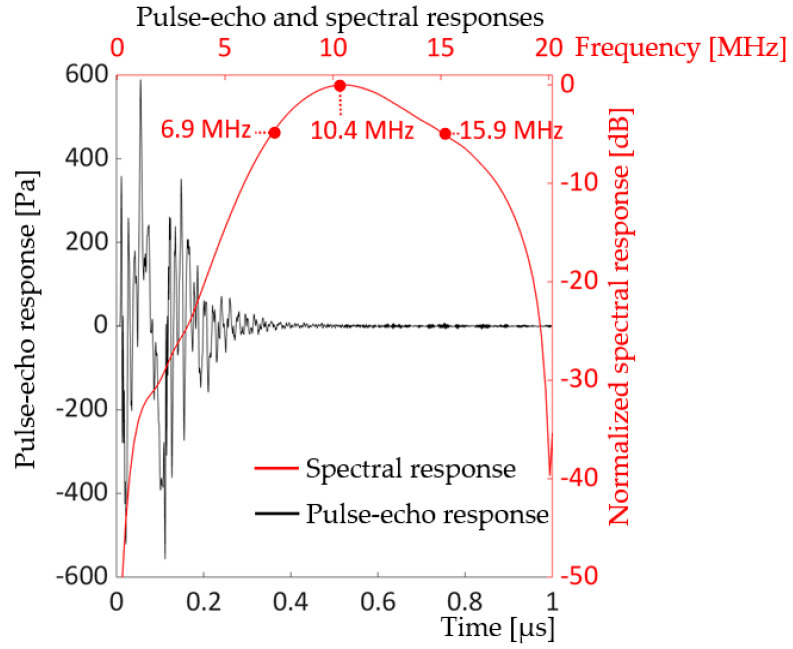
Electrical input impedance of the modified unit-cell under water load and air-backing driven by 5 V_p_, 10 MHz single cycle sine wave with 50 Ω resistor in the circuit.

Fundamental characteristics of the unit-cell as a transducer device were evaluated by pulse-echo and spectral response analyses. The analyses were performed using ECHO and Kirchhoff extrapolation techniques for computational efficiency in PZFlex [25] (p.127), [41]. The distance of the virtual rigid reflector was located 5.5 mm from the front surface of the unit-cell. The 5.5-mm distance was selected based on the average depth of the dermis and the subcutis, the second layer and the innermost layer of the skin, respectively, through which veins and arteries are distributed [42]. Figure 11 exhibits the pulse-echo and spectral response of the unit-cell model driven by a zero-to-peak bipolar voltage of 5V_p_, 10 MHz single cycle sine wave with 50 Ω resistor. The driving voltage was adjusted from the peak-to-peak bipolar 2V_pp_ for the vacuum model to 5V_p_ to be consistent with predefined experimental measurements of fabricated PMUT devices in which the maximum driving voltage amplitude was set to 5V_p_, zero-to-peak, either bipolar or unipolar.

In Figure 11, the center frequency was 10.4 MHz, very close to the frequency goal of 10 MHz. The difference, 0.4 MHz, is thought to arise from the PI thickness adjustment made earlier for mass-loading compensation. Lower and upper −6 dB frequencies were 6.9 and 15.9 MHz, respectively, yielding a bandwidth of 86.5 % and quality factor of 1.16. The wide bandwidth and low quality-factor indicates that the unit-cell would have a good receiving performance and be suitable for pulse-echo imaging.

#### 2.3.3. Mode Shape Profiles

**Figure 12:**
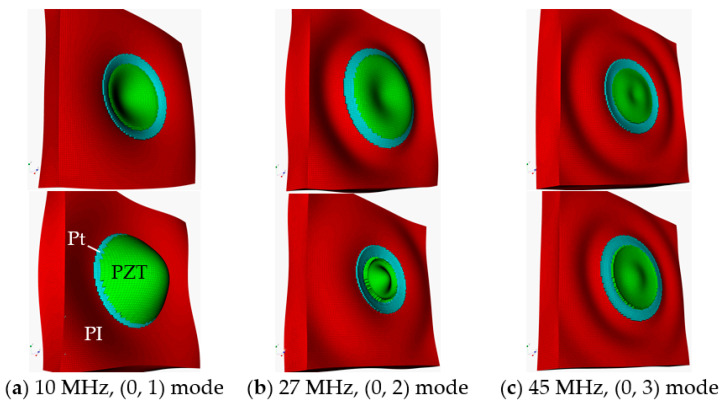
Mode shapes of the unit-cell model at (**a**) 10 MHz showing (0, 1) mode, (**b**) 27 MHz showing (0, 2) mode, and (**c**) at 45 MHz showing (0, 3) mode vibrations, driven by 10 MHz single cycle sine wave with 5 V_p_.

When a transducer is excited, more than one mode can be generated, and modes may be coupled to affect transducer performance. Mode shape profiling was carried out to discover potentially undesired modal distortion. The first three resonances, 10, 27, and 45 MHz, found in Figure 7, were selected for the modal analysis. Figure 12 shows mode shapes of the model at the three frequencies, which were driven by a zero-to-peak bipolar 5 V_p_, 10 MHz, single cycle sine wave. In Figure 12a,c, deflections of the diaphragm are depicted in the −Z direction (upper) and +Z direction (lower). In Figure 12a, the vibration of the unit-cell displays (0, 1) mode and the dominant motion of the unit-cell is in the Z direction. The diameter of the deflecting circle and the amplitude of the deflection are largest at 10 MHz among the three frequencies. In Figure 12b, at 27 MHz, both deflection amplitude and the area of the vibrating part at the center of the diaphragm decreased noticeably. The unit-cell shows (0, 2) mode vibration and guided waves on the surface of the PI substrate were observed. In Figure 12c, at 45 MHz, the unit-cell shows (0, 3) mode vibration and guided waves on the surface of the PI substrate. The size of the deflecting circle at the center and the amplitude of the deflection was smallest among the three.

Analyses revealed that, for higher resonances, there were more guided waves on the PI substrates and smaller deflections in the Z directions. Another important observation was that the PI substrate, not just its surface, deformed as the diaphragm vibrated in all three cases, which may result in compromised performance, especially bandwidth, of the sensor when extended to an array. This phenomenon will be discussed in Section 3, “Characterization of small arrays”.

## 3. Characterization of Small Arrays

**Figure 13:**
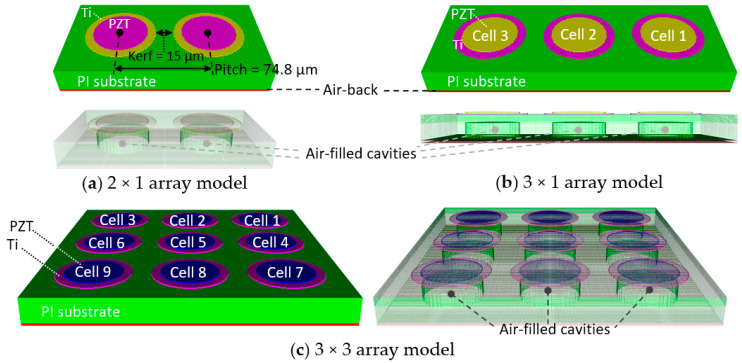
Schematics of the small arrays investigated. (**a**) 2 × 1, (**b**) 3 ×1, and (**c**) 3 × 3 arrays having the same kerf = 15 µm, the same pitch = 74.8, and the same unit-cell dimensions.

An investigation of how PI substrate deformation and the appearance of guided waves on the surface can affect the characteristics of an array was carried out for 2 × 1, 3 × 1, and 3 × 3 array models prior to developing a full 16 × 16 array model. Displacements of the unit-cells were plotted against time, and the synchronization of the array was assessed. The model center frequencies and bandwidths were compared. Figure 13 displays schematics of the small array models, all of which have 25 °C water as the front load medium with the same boundary conditions as depicted in Figure 10.

In Figure 13, all three array models consisted of the unit-cells in Figure 8 and had the same kerf and pitch of 15 and 74.8 µm, respectively. The cavities were filled with air and the bottoms were backed with a 2-µm air layer.

### 3.1. Displacement Synchronization and Oscillation Decay

**Figure 14:**
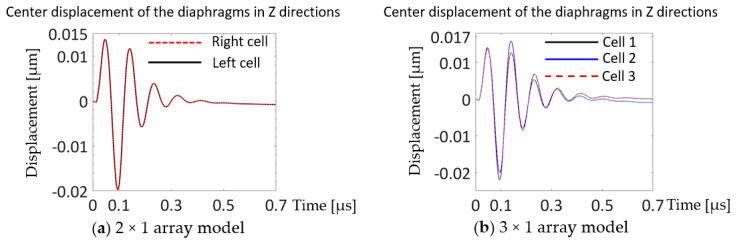
Z displacement of the cells at their centers in the (**a**) 2 × 1 and (**b**) 3 × 1 array models. Both driven by zero-to-peak bipolar 5 V_p_, 10 MHz, single cycle sine wave with 50 Ω resistor in the circuit.

Figure 14 displays time histories of motions in the Z-directions of the cells for the 2 × 1 and 3 × 1 array models.

**Figure 15:**
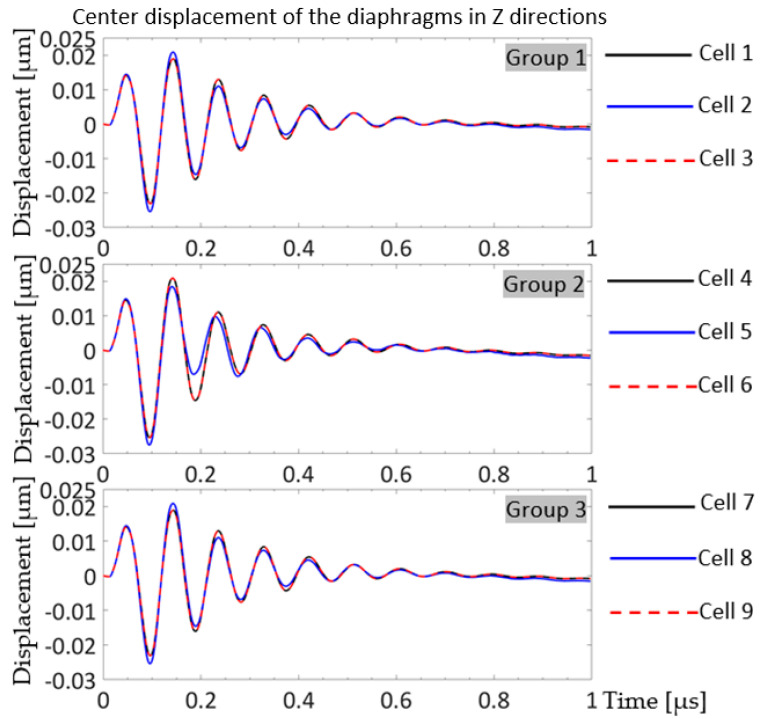
Z displacement of the cell centers in the 3 × 3 array model driven by 5 V_p_, 10 MHz, single cycle sine wave with 50 Ω resistor in the circuit.

In Figure 14a, the two cells show exactly the same movement in the Z-directions, and four and a half decay cycles. In Figure 14b, edge cells in Figure 14b, cell 1 (solid black curve) and cell 3 (dashed red curve) have the same displacements in the Z-directions as a function of time, while the center cell, cell 2 (blue curve) shows larger peak amplitude in the first one and a half cycles and starts showing smaller peak-to-peak amplitudes than the other two cells from the first cycle. Cell 2 showed its largest positive peak on the second cycle while cell 1 and cell 3 damped after the first oscillation cycle. A greater number of oscillation decay cycles were observed in the 3 × 1 array than in the 2 × 1 array in Figure 14. Figure 15 shows the time history of the Z displacements of cells in the 3 × 3 array model.

In Figure 15, the center displacement of the cells is displayed in three groups: from the top, group 1 with cells 1, 2, and 3; group 2 with cells 4, 5, and 6; and group 3 with cells 7, 8, and 9. The cell numbers are labeled in Figure 13c. All the cells in the 3 × 3 array model decayed after approximately eight oscillation cycles, taking a larger number of cycles and longer times than the 2 × 1, and 3 × 1 models. Furthermore, their first +Z-direction peaks were smaller than their second +Z-direction peaks, as observed for the middle cell of the 3 × 1 array model in Figure 14b. The displacements of group 1 and group 2 show the same trend—the cells positioned in the middle, cell 2 and cell 8, of the rows displayed larger amplitudes at the first peak in the –Z-direction and at the second peak in the +Z-direction, and then exhibited smaller peaks in both directions for the next three cycles until their motions corresponded to the edge cells of their rows. The incoherence of cell 5, the middle cell, in group 2 was more noticeable, since cell 5 was positioned at the center of the array and its motion could be influenced by all eight cells around it. The models with a higher number of cells had larger displacement amplitudes, more decay cycles, longer ring-down in pulse-echo responses, and eventually narrower system bandwidths. In the following section, pulse-echo and spectral responses will be observed and center frequencies and bandwidths of the models will be compared.

### 3.2. Pulse-Echo and Spectral Responses

**Figure 16:**
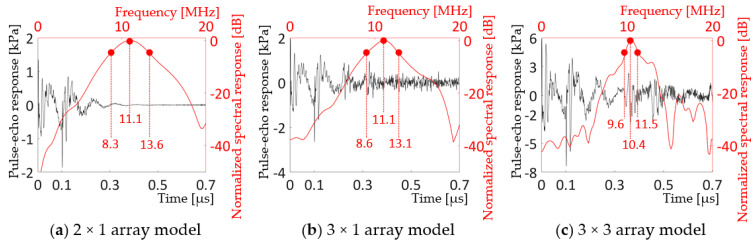
Pulse-echo and normalized spectral response of the (**a**) 2 × 1, (**b**) 3 × 1, and (**c**) 3 × 3 array models driven by 5 V_p_, 10 MHz, single cycle sine wave with 50 Ω resistor in the circuit.

Figure 16 shows the pulse-echo and normalized spectral responses of the 2 × 1, 3 × 1, and 3 × 3 array models. In Figure 16, longer ring-down and noisier pulse-echo responses were found as the number of cells increased in the models. Table 2 compares the center frequencies and bandwidths of the small array models and the unit-cell model.

In Table 2, a 2 × 2 array model was added for comparison even though its analyses were not included in the text. Table 2 reveals that the bandwidth tends to decrease as the number of cells increases, although the bandwidth of the 2 × 2 array was 0.7% wider than that of the 3 × 1 array. The center frequencies of the models indicated that *m* × *m*, symmetric array models had center frequencies very close or equal to that of the unit-cell, while *m* × *l*, asymmetric array models had increased center frequencies. Further research is required to determine the effect of mechanical constraint on the center frequencies in the symmetric versus asymmetric array models.

### 3.3. PI Substrate vs. Si Substrate

The analyses suggested that bandwidth could be significantly reduced as the number of cells increased due to the longer oscillation decay cycles of individual cells, and thus prolonged ring-down of the echo. The compliance of the PI substrate could explain these phenomena. Superposed waves from multiple vibration sources cause larger amplitude and more cycles of oscillation in the compliant substrate. To explore this deduction, the same unit-cell and small array models with Si substrates were simulated and compared with the values in Table 2. Si has an elastic modulus varying from 130.2 GPa to 187.5 GPa [43], which is at least 15 times larger than that of PI, 8.5 GPa [28]. Table 3 displays Si substrate models in terms of center frequency and bandwidth.

In Table 3, the Si substrate models with the same geometries have higher center frequencies than those for the PI substrate models in Table 2, results explained by the difference in their elastic moduli and the inability to extend the effective area of the vibrating diaphragm. The higher center frequency of the Si substrate resulted in narrower bandwidths for the unit-cell models compared to the PI substrate unit-cell models. Both Si and PI substrate models exhibited a decrease in bandwidth as the number of cells increased (Table 3 and Table 4). From unit-cell to 3 × 3 models, the bandwidth of the Si substrate group was reduced by 51%, while that of the PI substrate group showed a 78.8% reduction. Table 3 also suggests that the bandwidth decrease for the Si substrate correlated more with the increase in center frequencies than the narrower distance between the lower and upper -6 dB values, as the number of cells increased.

Attenuation coefficients, another important material property, were considered. Table 4 shows the attenuation coefficients used for both PI and Si substrates models.

The attenuation coefficients of PI were calculated from its density and bulk and shear acoustic velocities, 1082 kg/m^3^ [28], and 3500 and 2000 m/s [44], respectively, using the Damping Tool in PZFlex with viscoelastic damping model [29] (p.89). The Si attenuation coefficients were taken from the PZFlex Materials Database. In Table 4, PI has bulk and shear attenuation coefficients 90 and 43 times greater than those of Si, respectively. While a larger attenuation coefficient means more effective damping of vibrations and wave propagation, the PI substrate models revealed a higher number of decay cycles and narrower bandwidths as the number of cells increased. This was probably because the PI material was part of the vibrating structure, rather than being a damping backing module.

The pulse-echo and spectral responses and the comparison analyses suggested that the compliance of the PI material was the main cause of the bandwidth reduction. Different designs with a PI substrate were studied, but no successful case of preventing the bandwidth reduction was discovered. Attempted designs included: (1) placing soft backing material, Vantico HY956/CY208 with an elastic modulus of 1.998 GPa [27], in the cavity and beneath the PI substrate to reduce reflection from the back; (2) placing a rectangular trench around the array to prevent reflections from the edges to the cells; and (3) employing trenches around each cell to reduce interferences between cells. These phenomena were thought to be trade-offs for using a flexible substrate. However, it became apparent that, for a full 16 × 16 array, the unit-cell design must achieve greater displacement of the diaphragm to accommodate the high attenuation of human tissues [45,46], i.e., 0.54 dB/(MHz·cm) for soft tissue, which is orders of magnitude greater than that of water, 0.0022 dB/(MHz·cm), instead of widening the bandwidth.

## 4. 16 × 16 2D Array

A 2D array, consisting of 256 (16 × 16) cells, was created and characterized in terms of surface displacement, pulse-echo and spectral responses, beam profile, and crosstalk. The unit-cell of the array was determined to have the maximum diaphragm displacement throughout a series of case studies. The first case study began with the unit-cell (7-µm thick and 46-µm diameter diaphragm) shown in Figure 8. The unit-cell was modified to have a thinner and smaller diaphragm, 2 µm thickness and 31.96 µm diameter, to have greater diaphragm displacement and more space between adjacent cells. The modified unit-cell showed improved peak-to-peak displacement, 0.068 µm, from that of the initial unit-cell, 0.029 µm, when driven by a 5 V_p_, 10 MHz, single cycle sinewave with 50 Ω resistor in the circuit. The second case study explored varied combinations of the PZT active layer, Ti passive layer, and cavity diameters: (1) the same diameter, 31.96 µm, for active layer and cavity, and 30% larger passive layer diameter; (2) the same diameter, 31.96 µm, for all the three layers; and (3) the same diameter, 31.96 µm, for passive layer and cavity, and 30% smaller active layer. The results gave their maximum peak-to-peak diaphragm displacements, from case (1) to case (3), of 0.083, 0.097, and 0.068 µm, respectively, when driven by zero-to-peak bipolar 5 V_p_, 10 MHz, single cycle sinewave with 50 Ω resistor in the circuit. The third case study varied the top electrode area; it was fixed to be either the same as the PZT active layer; and 90%, 80%, 70%, or 60% of the PZT diameter. The results showed that the unit-cell diaphragm achieved the maximum displacement when all the layers, from the top, top electrode, PZT active layer, bottom electrode, Ti passive layer, and cavity, had the same diameter. These results differed from those previously reported by Choi et al. [47], Yaacob et al. [48], and Sammoura et al. [49] and were attributed to the use of flexible PI substrates.

### 4.1. Construction of the 16 × 16 Array

A 2D array was constructed based on the above case studies. Figure 15 shows top and cross-section views of the 2D array model.

**Figure 17:**
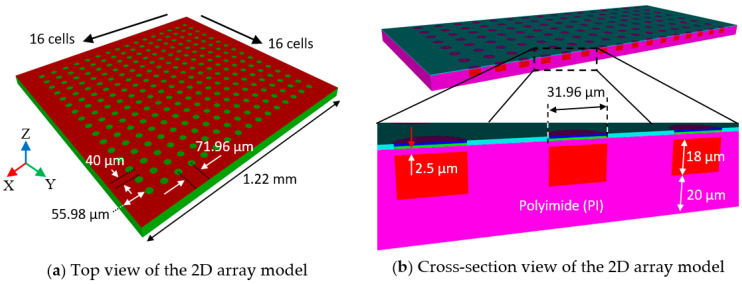
(**a**) top view and (**b**) cross-section view of the 2D array model with dimensions.

In Figure 17a, the pitch, i.e., the center-to-center distance between cells, is 71.96 µm, and the kerf, i.e., the edge-to-edge distance between cells, is 40 µm. The 2D array has an edge width, 55.98 µm, on all four sides and side length of 1.22 mm, making its total area approximately 1.5 mm^2^. The ultrasound launching direction is +Z direction, perpendicular to the surface of the cells. The magnified cross-section view in Figure 17b shows the thickness of the PI passive layer, the air-filled cavity and the PI substrate to be 2.5, 18, and 20 µm, respectively. The PZT active layer (blue), Ti (light blue) passive layer, and PI passive layer were determined to have the same diameter, 31.96 µm, to achieve the maximum diaphragm deflection as described above. The thicknesses and the diameter were defined to have the center frequency near 10 MHz. Quarter symmetry was applied to build the whole array. The 2D array model was then characterized in terms of surface displacement profile, pulse-echo and spectral responses, beam propagation profile and crosstalk.

### 4.2. Surface Displacement Profile

**Figure 18:**
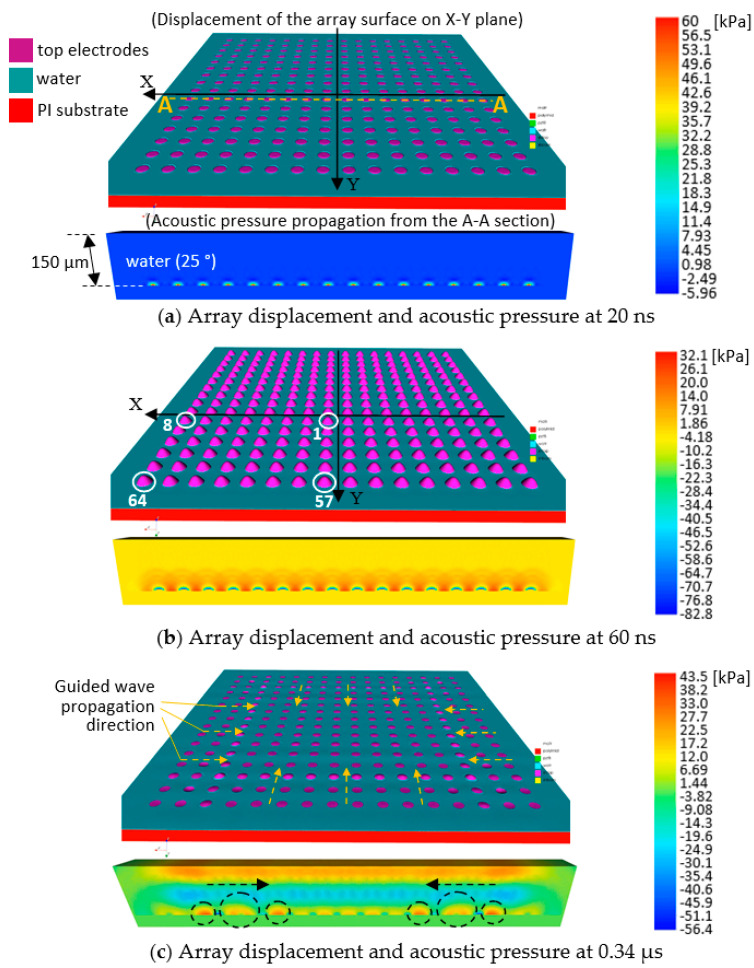
Snapshots of the 10 MHz 2D array model at (**a**) 20 ns, (**b**) 60 ns, and (**c**) 0.34 µs, driven by zero-to-peak bipolar 5 V_p_, single cycle sine wave with 50 Ω resistor in the circuit, displaying array displacement and acoustic pressure propagation.

Synchronization of the cells and undesirable guided wave effects were inspected through surface displacement profiling of selected cells. Figure 18a–c display snapshots of plane and cross-section views of the array model, highlighting the acoustic pressure field and its propagation at different time frames, 20, 60, and 0.34 µs, respectively.

**Figure 19:**
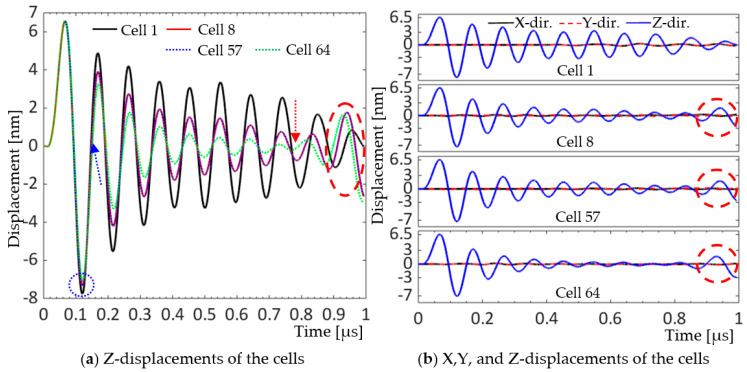
Displacements of the selected cells (**a**) in Z-direction, and (**b**) in X-, Y-, and Z-directions at their centers.

The model was driven by zero-to-peak bipolar 5V_p_, 10 MHz, single cycle sine wave with 50 Ω resistor in the circuit. In Figure 18a, a quarter symmetry model was built in the X-Y plane. The A-A section passed through the cell centers in the first row along the X axis, with rows increasing in the +Y direction. Figure 18a–c show the acoustic pressure field formed from the A-A section and propagating in the 150-µm thick water load, approximately a wavelength of the ultrasound in 25 °C water, in front of the array at the corresponding time frame. The color bars display the pressure amplitude in the corresponding cross-sections; + sign, and − sign indicate tensile and compressive pressures, respectively. In Figure 18a, the motion of the cells and acoustic pressure each cell generated, 20 ns after activation, was observed. In Figure 18b, at 60 ns, all the cells deformed upward coherently and spherical waves from the cells can be seen propagating into the water on the cross-section view. Up to approximately 0.1 µs, the movement of the cells were well synchronized, and the spherical waves combined forming a horizontally long plane wave propagating in the water. The highlighted cells in Figure 18b—cell 1, one of the four center cells of the array, cell 8 and cell 57, cells in the middle of the edges, and cell 64, a corner cell—were selected to plot their displacements versus time. In Figure 18c, at 0.34 µs, the propagation of the guided waves, inward from the edges, is clearly visualized in both the X-Y plane view and the cross-section view, as marked with dashed black circles. Figure 19 compares the displacements of the centers of the selected cells in Figure 18b.

In Figure 19a, the four cells synced well in the first half cycle in terms of both displacement amplitude and phase. In the second half cycle, at 0.12 µs, indicated by the dotted blue circle, they exhibited small amplitude differences at their peaks. From the beginning of the second cycle, at about 0.15 µs, indicated by the dotted blue arrow, they began to have subtle differences in phase and greater amplitude differences in the subsequent positive and negative peaks. Cell 8 and cell 57, the middle cells on the edges, had identical movements with time. The amplitude difference grew or maintained up to approximately 0.78 µs, indicated by the dotted red arrow, beyond which the differences in the phase became large. Approximately from 0.88 µs, cells 8 and 57, and cell 64 revealed increased displacement amplitudes as shown by the dotted red oval in Figure 19a and the dotted red circles in Figure 19b, which are strong indications of guided wave propagation. Figure 19b showed that displacements of the four cells were dominant in the Z-direction and negligible in X and Y directions and suggested that the guided wave propagation did not affect motions of the cells in horizontal directions but in vertical directions.

### 4.3. Pulse-Echo and Spectral Responses

**Figure 20:**
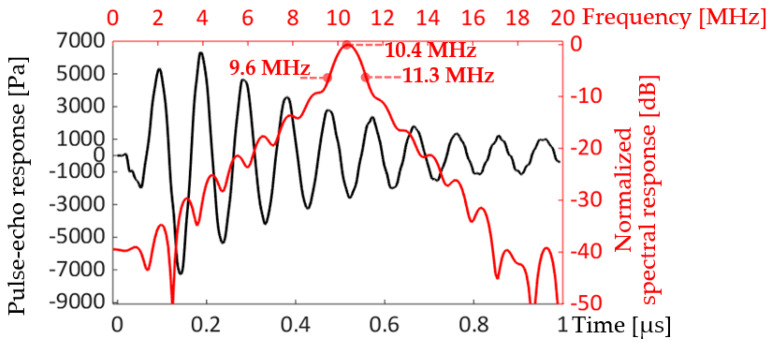
Pulse-echo (black plot) and spectral responses (red plot) of the 16 x 16 2D array.

Figure 20 shows the calculated pulse-echo and spectral responses for the 16 x 16 2D array model.

The pulse-echo response in Figure 20 was calculated with a virtual reflector located 5.5 mm ahead of the array surface. The pulse-echo response had a long ring-down time and multiple cycles before the echo decreased to zero. The spectral response, consequently, shows a narrow bandwidth, 16%, from the lower and upper -6 dB frequency points, 9.6 and 11.3 MHz, respectively. The bandwidth decreased dramatically from the 86.5% of the unit-cell model, shown in Figure 11 and Section 3, “Characterization of small arrays”’

### 4.4. Beam Propagation Profile

**Figure 21:**
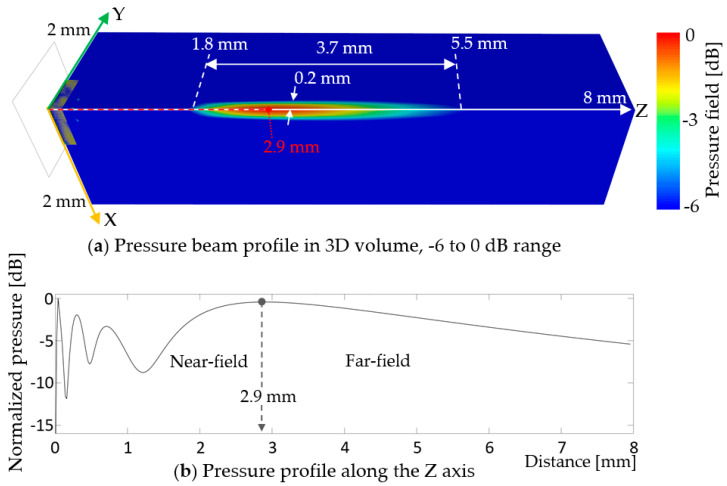
Beam profile of the 2D array in (**a**) -6 dB range and (**b**) pressure profile along the Z axis.

Beam profile modeling visualizes ultrasound wave propagation in the medium, water at 25 °C. Near-field distance, depth of focus, and side lobes can be evaluated. Figure 21 shows the wave propagation of the 2D array model in the −6 to 0 dB range on the quarter block of the model, and the pressure profile along the Z axis.

In Figure 21a, the acoustic waves focus in a thin elliptical shape from 1.8 to 5.5 mm, providing the depth of field, 3.7 and 0.2 mm radius at its thickest part. The focal length is found 2.9 mm from the surface of the array, which coincides with the near-field/far-field boundary in Figure 21b. In the -6 dB range, no side lobes were observed. The 3D beam profile indicated that the design would provide good penetration depth, sharp focus, and no concerns regarding side lobes.

### 4.5. Crosstalk

**Figure 22:**
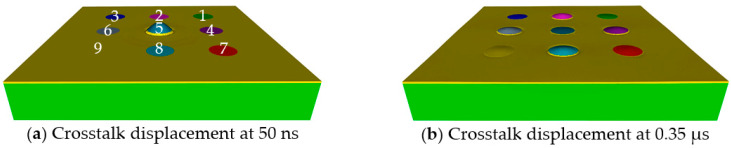
3 × 3 array model used for crosstalk analysis at times of (**a**) 50 ns and (**b**) 0.35 µs.

Crosstalk is an interference phenomenon of adjacent cells, which can potentially degrade the performance of the entire array. The designed array was expected to experience crosstalk due to the compliance of the PI substrate and the absence of kerf material. Crosstalk modeling requires all the individual cells to be connected to individual top and bottom electrodes, and individual electrical circuits, so that the electrical response of each cell can be calculated in PZFlex. Since such calculations require a heavy computation load and elongated analyses times, the electrical crosstalk was evaluated in a reduced, 3 × 3 array model with the same design parameters as the 16 × 16 array. Figure 22a,b display the 3 × 3 array model used for crosstalk analysis at times of 50 ns and 0.35 µs, respectively, all of which have 25 °C water-load in front with the same boundary conditions depicted in Figure 10.

**Figure 23:**
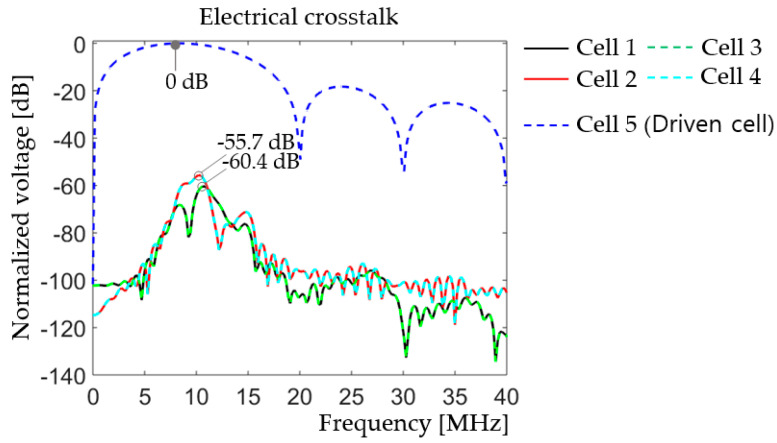
Electrical crosstalk of the 3 × 3 array showing adjacent cell voltage outputs normalized by that of the driven cell (Cell 5) in the frequency range 0 – 40 MHz.

In Figure 22, cell 5 is the only driven cell at 10 MHz single cycle sine wave with 5 V_p_ amplitude. Electrical responses of adjacent cells, cells 1, 2, 3 and 4, were monitored to evaluate crosstalk of the array. Figure 22a shows deformation of cell 5 at 50 ns after excitation, in which no other cells deformed. In Figure 22b, after 0.35 µs of the excitation of cell 5, deformations of the other cells are observed. Two groups of cells, middle cells (cells 2, 4, 6, and 8) and corner cells (cells 1, 3, 7, and 9), were found to have different phases depending on their distance from the driven cell. This phenomenon suggests spherical spreading of guided waves on the surface of the array. Figure 23 displays the electrical crosstalk of the model.

In Figure 23, the two corner cells (cells 1 and 3) show the same voltage response −60.4 dB below that of the driven cell, cell 5. The two cells in the middle of the edges (cells 2 and 4) display the same voltage response −55.7 dB below that of cell 5. The maximum normalized voltage response, −55.7 dB, of the adjacent cells is well within the acceptable range, i.e., below −30 dB [50], and indicates that the model will not have crosstalk issues.

## 5. Discussion

Comprehensive finite element modeling was carried out to design a thin-film, PZT-based, 10 MHz, 2D transducer array. A prototype unit-cell composed of concentric layers of, from the top, 1-µm thick, 46-µm diameter PZT active layer, sandwiched between 50-nm thick, 46-µm diameter top and bottom Pt electrodes; 1-µm thick, 59.8-µm diameter Ti passive layer; 7-µm thick, 46-µm diameter PI passive layer; and 13-µm deep, 46-µm diameter air-filled cavity with a PI substrate, gave a resonance frequency at 10.4 MHz and 86.% bandwidth in water. The analyses of small, 2 × 1, 3 × 1, 2 × 2, and 3 × 3 array models, consisting of the unit-cells, showed a dramatic decrease in bandwidth, from 86.5% for the unit-cell to 47.7%, 41.0%, 41.7%, and 18.3% for the 2 × 1, 3 × 1, 2 × 2, and 3 × 3 arrays, respectively. A comparison study with rigid Si substrate models indicated that the reduction in bandwidth originated from the compliance of the PI substrate. A new unit-cell design was found for the full-size, 16 × 16, 2D array to achieve larger deflection of the diaphragm and give larger kerf to lessen the interference between the cells. It consisted of, from the top, concentric layers of: 50-nm thick Pt electrode; 1-µm thick PZT active layer; 50-nm thick Pt electrode; 1-µm thick, Ti passive layer; 2.5-µm thick, PI passive layer; and 18-µm deep, air-filled cavity; all with the same 31.96 µm diameters on a PI substrate. The multi-domain finite element characterization revealed that the thin-film, PZT-based PMUT 2D array had 10.4 MHz center frequency, -55.7 dB crosstalk, and for -6 dB range: bandwidth, depth of field, and mechanical focal length of 16%, 3.7 mm from 1.8 to 5.5 mm in a thin elliptical shape, and 2.9 mm, respectively. These results suggest that the flexible PMUT 2D array would have good penetration depth with sharp focus and directionality, acceptable crosstalk, but narrow bandwidth.

## 6. Conclusions

This study showed that closed-loop finite element analyses play a key role in finding robust designs for high-frequency ultrasound devices. A virtual-prototype was built and its performance was predicted through: (a) pulse-echo and spectral responses to determine center frequencies and bandwidths; (b) surface displacement profiles to show synchronization of cells and find undesirable guided wave effects; (c) mode shape profiling to discover unwanted modal distortions; (d) beam propagation profiling to visualize ultrasound propagation and focusing; and (e) crosstalk modeling to diagnose whether the interference between cells was acceptable. The virtual prototyping identified robust designs which could potentially reduce the time and money required for fabricating and testing multiple prototypes. Such comprehensive modeling studies provide important guidance for future ultrasound device research and development.

## Figures and Tables

**Table 1:** Mechanical and damping properties of the 10 MHz circular unit-cell prototype 1 in Figure 2.

Material	Mechanical			Damping (Viscoelastic)	
	Density	Bulk Velocity	Shear Velocity	Bulk Attenuation	Shear Attenuation
Pt	21,400 kg/m^3^	3260 m/s	1730 m/s	0.3 dB/MHz/cm	0.9 dB/MHz/cm
Ti	4480 kg/m^3^	6100 m/s	3100 m/s	0.3 dB/MHz/cm	1.2 dB/MHz/cm
PI	1082 kg/m^3^	3500 m/s	2000 m/s	9 dB/cm at 10 MHz	13 dB/cm at 10 MHz

**Table 2:** Center frequencies and bandwidths of the small array models and the unit-cell model.

		Unit-Cell	2 × 1	3 × 1	2 × 2	3 × 3
**PI substrate**	Low/Upper -6 dB [MHz]	6.9/15.9	8.3/18.4	8.6/13.1	8.6/13.1	9.6/11.5
	Center freq. [MHz]	10.4	11.1	11.1	10.8	10.4
	Bandwidth (−6 dB)	86.5%	47.7%	41.0%	41.7%	18.3%

**Table 3:** Center frequencies and bandwidths of Si substrate small array and unit-cell models.

		Unit-Cell	2 × 1	3 × 1	2 × 2	3 × 3
**Si substrate**	Lower/Upper -6 dB [MHz]	9.2/17.8	9.7/18.4	8.6/18.6	11.5/18.4	13.6/18.7
	Center freq. [MHz]	13.8	15.1	14.9	15.3	16.7
	Bandwidth (−6 dB)	62.3%	57.6%	67.1%	45.1%	30.5%

**Table 4:** Attenuation coefficients applied to PI and Si substrate models at 10 MHz.

	PI	Si
Bulk attenuation coefficients	9 dB/cm	0.1 dB/cm
Shear attenuation coefficients	13 dB/cm	0.3 dB/cm
